# Neural and behavioural state switching during hippocampal dentate spikes

**DOI:** 10.1038/s41586-024-07192-8

**Published:** 2024-03-13

**Authors:** Jordan S. Farrell, Ernie Hwaun, Barna Dudok, Ivan Soltesz

**Affiliations:** 1 Department of Neurosurgery, Stanford University, Stanford, CA USA; 2 F.M. Kirby Neurobiology Center and Rosamund Stone Zander Translational Neuroscience Center, Boston Children’s Hospital, Boston, MA USA; 3 Department of Neurology, Harvard Medical School, Boston, MA USA; 4 Departments of Neurology and Neuroscience, Baylor College of Medicine, Houston, TX USA

## Abstract

Distinct brain and behavioural states are associated with organized neural population dynamics that are thought to serve specific cognitive functions^1–3^. Memory replay events, for example, occur during synchronous population events called sharp-wave ripples in the hippocampus while mice are in an ‘offline’ behavioural state, enabling cognitive mechanisms such as memory consolidation and planning^4–11^. But how does the brain re-engage with the external world during this behavioural state and permit access to current sensory information or promote new memory formation? Here we found that the hippocampal dentate spike, an understudied population event that frequently occurs between sharp-wave ripples^12^, may underlie such a mechanism. We show that dentate spikes are associated with distinctly elevated brain-wide firing rates, primarily observed in higher order networks, and couple to brief periods of arousal. Hippocampal place coding during dentate spikes aligns to the mouse’s current spatial location, unlike the memory replay accompanying sharp-wave ripples. Furthermore, inhibiting neural activity during dentate spikes disrupts associative memory formation. Thus, dentate spikes represent a distinct brain state and support memory during non-locomotor behaviour, extending the repertoire of cognitive processes beyond the classical offline functions.

## Abstract (web summary)

The hippocampal dentate spike, an understudied population event that frequently occurs between sharp-wave ripples, may underlie a mechanism re-engaging the brain with the external world during synchronous population events, and support memory during non-locomotor behaviour.

## Main

During awake immobility and non-rapid-eye-movement sleep, hippocampal neural activity is compressed at irregular intervals into brief, synchronous population events marked by large amplitude local field potential (LFP) activity^13^. These synchronous hippocampal events come in two main types, defined by their distinct, subregion-specific dynamics. In the CA1, sharp-wave ripples (SPW-Rs) predominate and are identified by 20–200 ms of roughly 150 Hz oscillatory activity at the pyramidal cell layer and a wave of depolarization at the apical dendrites^1^. In dentate gyrus, short duration (less than 50 ms), large amplitude (greater than 1 mV) spikes are recorded in the hilus and are termed dentate spikes (DSs)^12^. SPW-Rs and DSs occur at similar rates of up to 0.5 Hz during low arousal brain states, but are non-overlapping at a subsecond timescale^12^, possibly owing to distinct brain and behavioural states. Moreover, SPW-Rs are thought to largely depend on intrahippocampal generation mechanisms^1^ whereas DSs are thought to rely on external input^12^, further underscoring distinct functional roles.

SPW-Rs are considered the most synchronous event in the mammalian brain and are associated with ‘offline’ memory replay in which neural activity is disengaged from the present setting to enable cognitive processes such as consolidation and planning. Whether DSs similarly reflect offline neural activity, accompany extra-hippocampal synchronous activity or serve a potentially distinct cognitive role is poorly understood^14–17^. Thus, our current understanding of possible neural computations during non-locomotor behaviour is limited to only one of the two main population patterns in the hippocampus. We sought to fill this gap by investigating the neural and behavioural dynamics during dentate spikes and assess its potential role in memory function.

## Distinct behavioural correlates of DSs

**Fig. 1:**
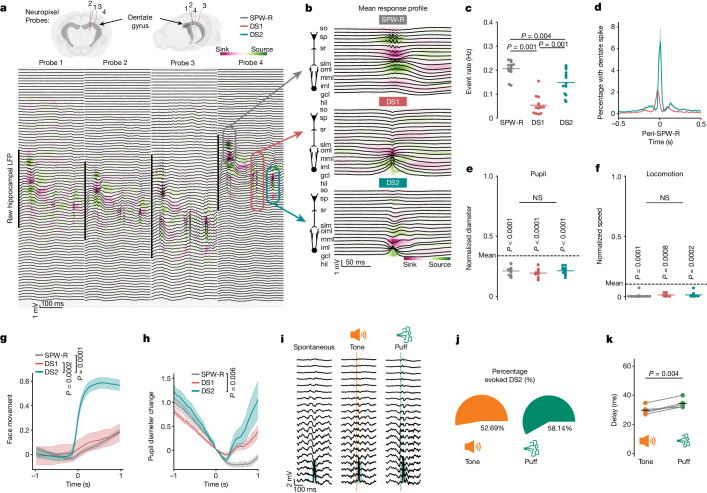
Distinct behavioural correlates of DSs. **a**,**b**, Raw LFP and CSD plots arranged from superficial to deep from four neuropixel probes intersecting different parts of the dentate gyrus. SPW-R (grey), DS1 (red) and DS2 (cyan) are denoted (**a**) and event-triggered averages for each event type are shown (**b**) in which cell cartoons denote pyramidal and granule cell layers. **c**, Incidence of each event from 13 recording sessions. Mean is represented by horizontal bars. Repeated measures one-way ANOVA: *F*_2,24_ = 68.65, *P* < 0.0001. Two-sided Tukey post test. **d**, Occurrence of DS1 and DS2 aligned to SPW-R incidence. **e**, Normalized pupil diameter during each event. Paired *t*-test, event mean versus across session mean (horizontal line). SPW-R, *t*_12_ = −9.78; DS1, *t*_12_ = −10.42; DS2, *t*_12_ = −9.58. No difference was observed between event types. Repeated measures one-way ANOVA: *F*_2,24_ = 3.19, *P* = 0.06. **f**, Normalized speed during each event. Paired *t*-test, event mean versus across session mean (horizontal line). SPW-R, *t*_12_ = −5.36; DS1, *t*_12_ = −4.48; DS2, *t*_12_ = −5.36. No difference was observed between event types. Repeated measures one-way ANOVA: *F*_2,24_ = 1.27, *P* = 0.46. **g**, Average (±s.e.m.) facial movement aligned to hippocampal LFP events. Repeated measures one-way ANOVA, *F*_2,4_ = 79.33, *P* = 0.0006. Two-sided Tukey post test. **h**, Average (±s.e.m.) pupil diameter change aligned to hippocampal LFP events. Data are the same as **e** but showing pupil diameter surrounding each event. Repeated measures one-way ANOVA, *F*_2,24_ = 8.35, *P* = 0.004. Two-sided Tukey post test. **i**, Representative spontaneous and evoked DS2. Vertical lines represent tone and puff onset. **j**, Pie charts show average percent of trials with evoked DS2 (*n* = 5). **k**, DS2 delay following tone or puff onset. Grey lines connect data points from the same mouse. Black lines are group means. Paired *t*-test (two-sided), *t*_4_ = −5.96. *n* = 5 mice. Data from **a**–**f** and **h** were obtained from the Allen Brain Institute^31^ (*n* = 13 mice), whereas **g** and **i**–**k** were obtained in the authors’ laboratory (Methods). gcl, granule cell layer; hil, hilus; iml, inner molecular layer; mml, middle molecular layer; oml, outer molecular layer; slm, stratum lacunosum moleculare; so, stratum oriens; sp, stratum pyramidale; sr, stratum radiatum.

**Extended Data Fig. 1:**
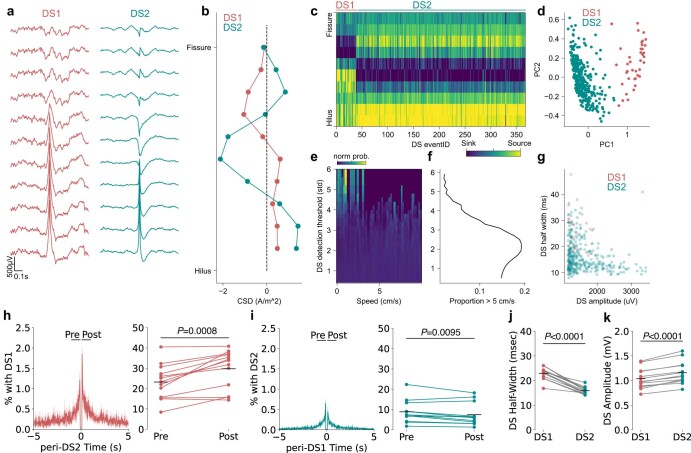
Classification of DSs from a representative recording session based on current source density (CSD) analysis. **a**, Event-triggered average of LFP traces for DS1 (in red) and DS2 (in blue). **b**, Average CSD depth profiles of DS1 (n = 39 events) and DS2 (n = 328 events). Voltage measurements for CSD analysis were taken at the peak of DS1 and DS2. Note that the maximal sink for DS2 locates deeper and hence closer to the hilus than the one for DS1. **c**, CSD depth profiles for all detected DS events, sorted by event types. **d**, Principal component analysis on CSD depth profiles. Each dot represents a detected DS event. Two clusters emerge following DBSCAN clustering, which correspond to two types of DS. **e**, Normalized probability distribution of detected DS events in various detection threshold and animal speed. Probability distribution is normalized within each level of detection threshold. **f**, False positive rate of detecting DS events. Detected DS events with animal speed over 5 cm/s were considered false positives. **g**, Amplitude and width of all detected DS events in the hilus. If multiple channels were in the hilus, the one with the maximal peak during DS was selected. Colors reflect the types of DS, as in **a**. **h**, DS1 events as a function of DS2 peak, expressed as the percent of total DS1 events occurring in the individual time bins. Right panel quantifies the total percent of DS1 events occurring within 1 second before (pre) or 1 second after (post) DS2. Paired t-test (two-sided), t_12_ = −4.48. **i**, same as **h** but for the reverse relationship with respect to DS1 and DS2. Paired t-test (two-sided), t_12_ = −3.08. **j**, Comparison of dentate spike half-width. Paired t-test (two-sided), t_12_ = 11.52. **k**, Comparison of dentate spike amplitude. Paired t-test (two-sided), t_12_ = 10.43. **a**–**g** are example data from the author’s lab. **h**–**k** are analyzed data (n = 13 mice) from the Allen Institute^31^.

**Extended Data Fig. 2:**
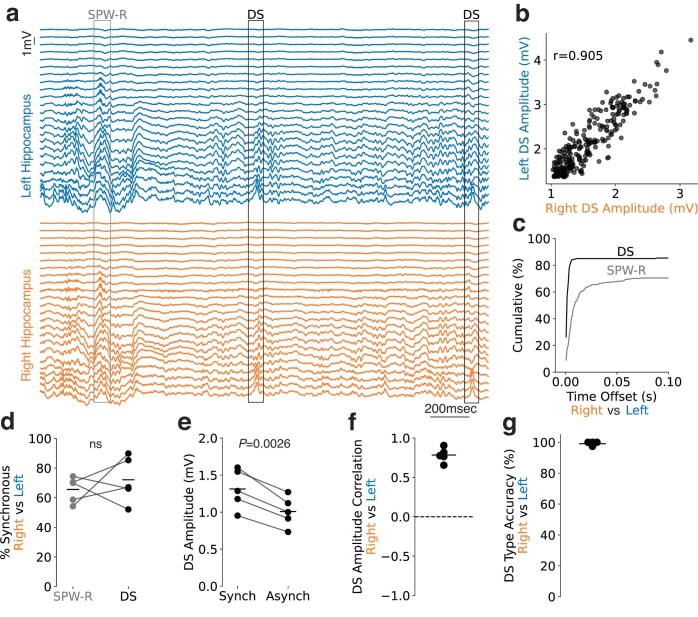
Bilateral synchrony during dentate spikes. **a**, Representative recording showing synchronous occurrence of dentate spikes (DS; black) and sharp-wave ripples (SPW-R; grey) in left (blue) and right (orange) hippocampi. Channels are organized by depth with superficial channels at the top of the page for each hemisphere. **b**, Amplitude of dentate spikes detected in each hemisphere from **a**. **c**, Cumulative distribution plot showing the temporal offset (interhemispheric inter-event interval) for DSs and SPW-Rs from **a**. **d**, Percent of events that are detected bilaterally within 100 msec. Connecting grey lines between points are from the same mouse. Horizontal lines are group means. Within-subject t-test (two-sided), t_4_ = 0.70, *P* = 0.52. **e**, DS amplitude difference between bilaterally synchronous (<100 msec) vs. asynchronous (>100 msec) events. Within-subject t-test (two-sided), t_4_ = 6.71. **f**, quantification of correlation values in **b** across all mice. **g**, quantification of event type (DS1 vs. DS2) concordance for synchronously detected DSs (<100 msec), showing that synchronous DSs are almost always the same type. Data were collected in the authors’ lab (n = 5 mice).

**Extended Data Fig. 3:**
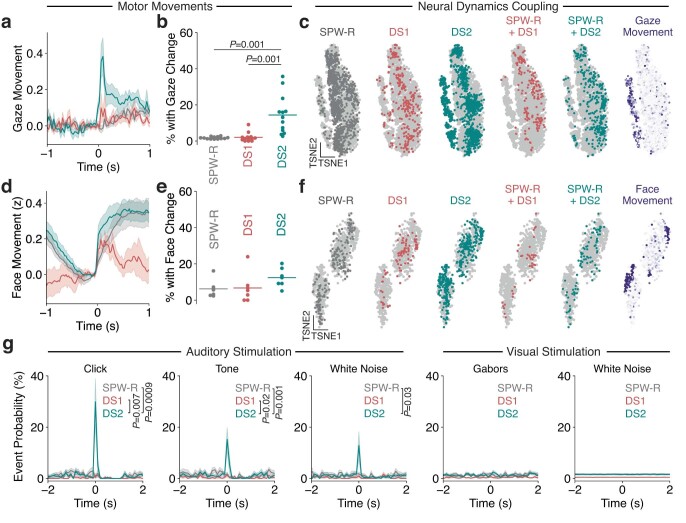
Coupling of type 2 dentate spikes to sensory input and motor behaviour. **a**, Average (±sem) changes in gaze speed (i.e., pupil movements) aligned to each hippocampal LFP event. **b**, Percent of events with an increase in gaze movement. Repeated measures one-way ANOVA F_2,24_ = 15.4, *P* < 0.0001. Two-sided Tukey Post-test. (n = 13 mice). **c**, Firing rate dynamics across all cells in a representative recording session during each LFP event (400 msec window) were projected onto two t-distributed stochastic neighbor embedding (TSNE) dimensions (grey background). Location of each event along these two dimensions are overlaid and colored according to event type/combination. Normalized gaze movement (right) during each event is plotted as color intensity, with purple being maximal. **d–f**, Same as **a–c**, but for face movements (n = 7 mice). Repeated measures one-way ANOVA F_2,12_ = 15.4, *P* = 0.10. **g**, Average (±sem) rate of occurrence of hippocampal LFP events aligned to trials of auditory or visual stimulation (n = 10 sessions). Repeated measures one-way ANOVA were performed on LFP event occurrence within 250msec of stimulus presentation versus an equal length of data prior to stimulus presentation. Click: F_2,18_ = 7.98, *P* = 0.003; Tone: F_2,18_ = 9.89, *P* = 0.001; White Noise (Aud): F_2,18_ = 4.30, *P* = 0.03; Gabors: F_2,18_ = 0.51, *P* = 0.61; White Noise (Vis): F_2,18_ = 0.12 *P* = 0.88. Two-sided Tukey post-test. Data in **a-c** are from the Allen Brain Institute^31^. Data from **d–g** are from the Steinmetz dataset^32^.

**Extended Data Fig. 4:**
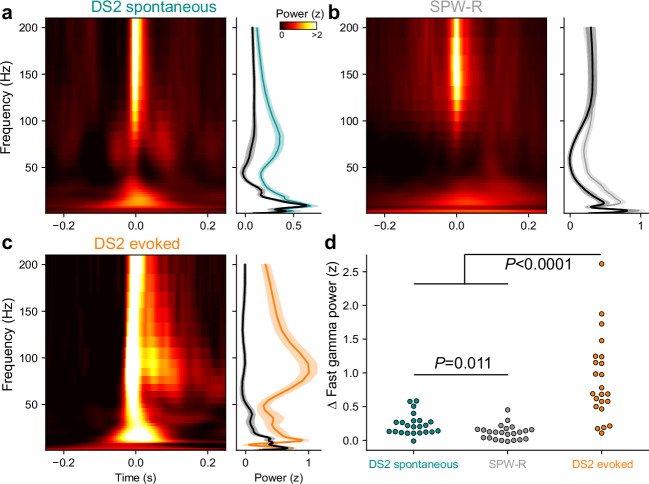
Selective elevation of fast gamma (65 − 120 Hz) power in DG following DS2. **a**, Average time frequency power spectrogram near spontaneous DS2 across mice from a recording channel located in the middle molecular layer of DG (n = 23 mice). The recording channel closest to the maximum sink from CSD depth profiles was chosen for each mouse. Color scale reflects normalized power, where the mean power of the entire recording session was used to compute z-scores. Mean power spectrograms before (from −0.15 s to −0.05 s) and after (from 0.05 s to 0.15 s) spontaneous DS2 were shown on the right panel in black and blue, respectively. Shaded regions indicate the standard error of mean. **b**, Same as in **a**, but for SPW-R. **c**, Same as in **a**, but for evoked DS2. **d**, Change in fast gamma power following spontaneous DS2, SPW-R, and evoked DS2 (n = 23 mice), Repeated measures one-way ANOVA F_2,44_ = 35.8, *P* < 0.0001, followed by pairwise two-sided multiple comparisons with Bonferroni correction. Each dot indicates one mouse.

**Extended Data Fig. 5:**
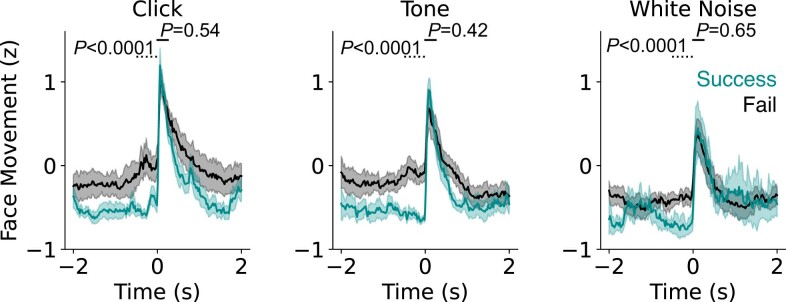
Behavioral state prior to auditory stimulation is associated with evoked DS2. Face movements (z-scored) are aligned to 3 different auditory stimulation onsets and displayed as mean ±sem. Trials are separated into those with successfully evoked DS2 (cyan) vs. no evoked DS2 (black) with stimulation. Changes in facial movement were analyzed before (−0.5 s to 0 s) and after (0 s to 0.25 s) auditory stimulation using a within-subject two-sided t-test. No differences in facial movements were observed after auditory stimulation at the peak of the motor response (Click, t_8_ = 0.64; Tone, t_8_ = −0.84; White Noise, t_8_ = −0.47), but significant differences were observed prior to stimulation (Click, t_8_ = 18.06; Tone, t_8_ = 52.51; White Noise, t_8_ = 34.98).

Using hippocampal electrophysiological recordings from awake, head-fixed mice (Fig. 1a) we first contrasted the behavioural dynamics during SPW-Rs and DSs. DSs were readily identified on the basis of their large voltage peaks in the hilus of the dentate gyrus (Fig. 1b) and further separated into the two previously identified types^12^ on the basis of current sinks in the outer (type 1, DS1) or middle (type 2, DS2) molecular layers (Fig. 1a,b and Extended Data Fig. 1). SPW-Rs were identified by transient increases in ripple band (120–180 Hz) activity in the CA1 pyramidal cell layer (Fig. 1b). Much like SPW-Rs, both DS1 and DS2 occurred synchronously across ipsilateral and bilateral recording sites (Fig. 1a and Extended Data Fig. 2). Consistent with previous research^1,12,18^, SPW-Rs and DSs occurred at similar rates (Fig. 1c, note that most DSs are DS2) during a low arousal state, marked by immobility and a relatively constricted pupil (Fig. 1e,f), and only a minority of DSs co-occurred with SPW-Rs (Fig. 1d). By examining behaviour at a subsecond timescale surrounding each event, we found that DS2 was tightly coupled with facial and eye movements (Fig. 1g and Extended Data Fig. 3a–f), followed by rapid pupil dilation (Fig. 1h) and increased hippocampal gamma power (Extended Data Fig. 4). Because this behavioural state was indicative of a brief and sudden arousal transition, we proposed that a startling sensory stimulus would also evoke DS2. Indeed, an auditory stimulus or air puff delivered to the face reliably evoked DS2 with short latency, but not DS1 or SPW-R (Fig. 1i–k and Extended Data Fig. 3g). There were no differences in the peak of the facial movement response on auditory stimulation for trials in which DS2 was successfully evoked compared to when they were not. Instead, trials with successfully evoked DS2 had lower facial movement activity before auditory stimulation (Extended Data Fig. 5), suggesting that a low arousal state may be an important precondition for DS2 rather than the magnitude of an evoked motor response.

**Extended Data Fig. 6:**
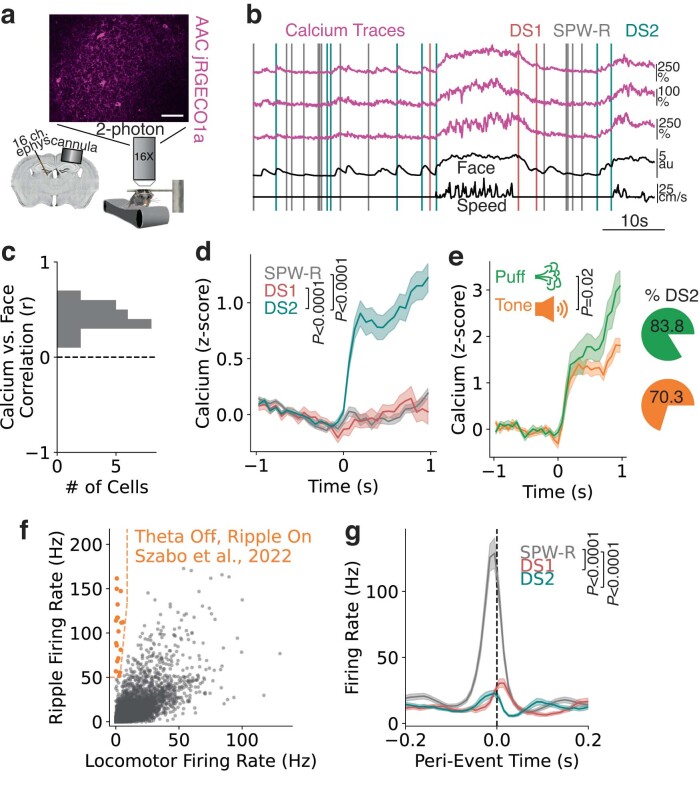
Selective activation of hippocampal GABAergic cells during DS2 vs. SPW-R. **a**, Calcium imaging of axoaxonic cells (AAC) was performed on awake behaving mice while contralateral electrodes captured hippocampal LFP events for panels. Image is averaged across movie frames from a representative recording (n = 3 mice). Scale bar = 50 μm. **b**, Representative calcium dynamics of 3 AACs during facial and locomotor movements. Hippocampal events are denoted by vertical bars. **c**, Histogram of correlation coefficients (r) for AAC calcium vs. facial movements (n = 25 cells, 3 mice). **d**, Average (±sem) AAC calcium activity aligned to hippocampal LFP events. Repeated measures one-way ANOVA, F_2,48_ = 38.32, *P* < 0.0001. Two-sided Tukey post-test. **e**, Average (±sem) AAC calcium changes aligned to air puff or tone onset. Two-sided within-subject t-test, t_24_ = 2.50. Pie charts show the percent of trials that evoke DS2 (n = 3 mice). Data were collected in the authors’ lab. **f**, Theta Off, Ripple On (TORO) cells identified in Szabo et al., 2022^21^ plotted in orange vs. all other cells recorded in the Allen Institute dataset. **g**, Event-triggered average firing rates of TORO cells for each event type. Repeated measures one-way ANOVA, F_2,34_ = 80.49, *P* < 0.0001.

One of the most arousal coupled cell types in the hippocampus is the GABAergic axo-axonic cell (AAC), whose activity we proposed would distinguish DS2 from DS1 and SPW-R. AACs, or chandelier cells, selectively innervate principal cell axon initial segments in the CA1 and are synchronously activated during arousal states, including facial movements during immobility, but not SPW-Rs^19,20^. Using two-photon calcium imaging of AACs (Unc5b-2A-Cre^ERT2^ mice expressing AAV1-FLEX-jRGECO1a), combined with silicon probe LFP recordings in awake behaving mice, we indeed found that AAC activity is correlated to spontaneous facial movements, increases in a time-locked manner to DS2, but not DS1 or SPW-R incidence, and increases during DS2-evoking sensory stimulation (Extended Data Fig. 6a–e). By contrast, the previously described GABAergic Theta Off Ripple On (TORO) cell^21^, which exclusively fires in high frequency bursts during immobility, is selectively active during SPW-Rs, but not DS2 (Extended Data Fig. 6f–g). These data further support that DS2 is a fundamentally distinct behavioural state, engaging orthogonal microcircuit elements within the hippocampus.

## Brain-wide activation during DS2

Because DS2 is associated with a unique behavioural state characterized by transient arousal bouts within a general low arousal state, we proposed that brain-wide activity patterns would reveal a distinct brain state from SPW-Rs. This is supported by previous research demonstrating that DSs are associated with more widespread cortical gamma synchrony than SPW-Rs during sleep^17^. Here, we used brain-wide surveys of action potentials from single units (that is, spiking neurons) in awake behaving mice to characterize the brain state that accompanies DS2 and compared this to SPW-R.

**Fig. 2:**
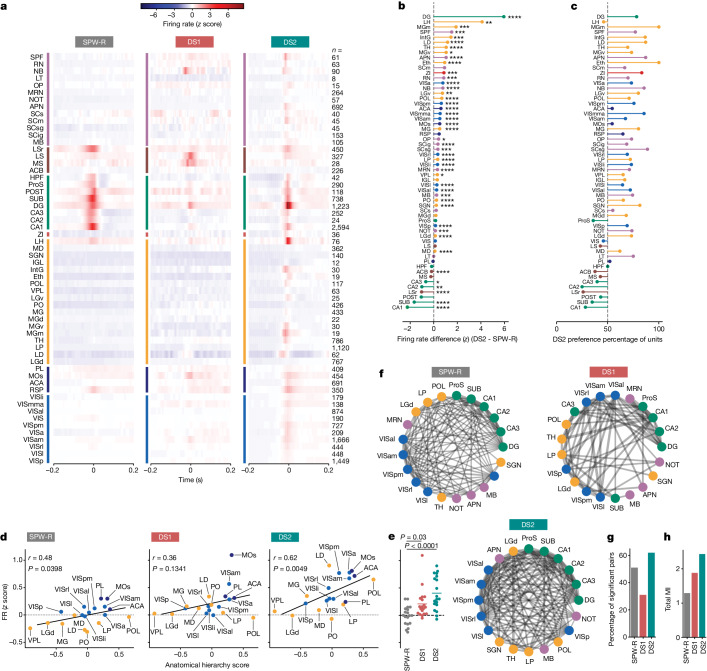
Elevated brain-wide firing during type 2 DSs. **a**, Event-triggered average firing rate expressed as a *z* score averaged across all cells at each recording location. **b**, Brain areas are sorted by the difference in average *z* scored firing rate change during DS2 versus SPW-R (*x* axis) and colour coded as in **a**. Within-subject *t*-tests (two-sided) were performed on each independent brain region to determine *P* values for *z* scored differences between SPW-R and DS2 across cells. *P < 0.05, **P < 0.01, ***P < 0.001, ****P < 0.0001. **c**, Proportion of units with a higher peak firing rate during DS2 than SPW-R, sorted as in **b** using the same data. **d**, Peak firing rate (*z* score) versus anatomical hierarchy score from ref. ^24^, analysed by linear regression (two-sided). Two-sided Pearson correlation coefficient (*r*) and *P* values shown for each event. **e**, Quantification of average peak firing rate, collapsed across brain areas in the anatomical hierarchy plot using the same *y* scale. Repeated measures one-way ANOVA: *F*_2,36_ = 22.62, *P* < 0.0001. Two-sided Tukey post test. *n* = 23 sessions from 20 mice. **f**, Mutual information between brain areas during hippocampal LFP events shown as a weighted connection, represented by grey line thickness. Only brain areas with significant coupling are linked by a line. Symbol colour corresponds to brain area groupings in **a**. **g**,**h**, Quantification of **f**. **g**, Percentage of brain region pairs with significant mutual information. **h**, Sum of mutual information for all significant pairs. Data are from the publicly available Steinmetz^32^ and Allen Brain Institute datasets^31^. See Extended Data Fig. 7 for abbreviations.

**Extended Data Fig. 7:**
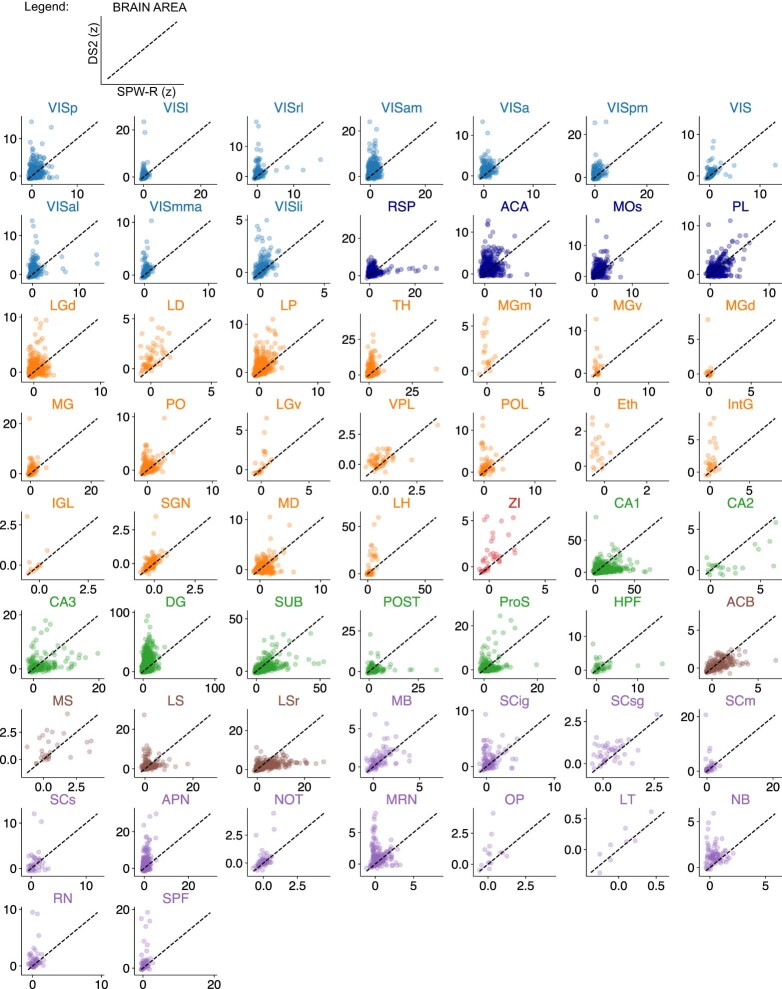
Firing rate changes during SPW-R and DS2 of individual units across brain areas. Legend shows x and y-axis labels for each panel. Brain areas are color-coded as in Fig. 2. Each dot is an individual cell. Data are the maximal z score for SPW-R vs. DS2. Note the off-diagonal pattern common to many brain areas. Data are from the Allen Brain Institute^31^ and Steinmetz datasets^32^. Abbrevations: For VIS = visual area; p=primary, l=lateral, rl=rostrolateral, am=anteromedial, a=anterior, pm=posteromedial, al=anterolateral, mediomedial anterior, li=laterointermediate. RSP=retrosplenial area. ACA=anterior cingulate area. MOs=secondary motor area. PL=prelimbic area. LGd=dorsal part of the lateral geniculate complex. LD=lateral dorsal nucleus of thalamus. LP= Lateral posterior nucleus of the thalamus. TH=thalamus. for MG=medial geniculate complex, m=medial part, v=ventral part, d=dorsal part. PO=posterior complex of the thalamus. LGv=ventral part of the lateral geniculate complex. VPL=ventral posterolateral nucleus of the thalamus. POL=posterior limiting nucleus of the thalamus. Eth=ethmoid nucleus of the thalamus. IntG=intermediate geniculate nucleus. IGL= intergeniculate leaflet of the lateral geniculate complex. SGN=suprageniculate nucleus. MD=mediodorsal nucleus of thalamus. LH=lateral habenula. ZI=zona incerta. For CA=Ammon’s horn, 1,2, and 3 are the respective fields. DG=dentate gyrus. SUB=subiculum. POST=postsubiculum. ProS=prosubiculum. HPF=hippocampal formation. ACB=nucleus accumbens. MS=medial septum. LS=lateral septum. LSr=rostral (rostroventral) part of the lateral septum. MB=midbrain. For SC=superior colliculus, ig=motor related intermediate gray layer, sg=supreficial gray layer, m=motor related, s=sensory related. APN=anterior pretectal nucleus. NOT=nucleus of the optic tract. MRN=midbrain reticular nucleus. OP=olivary pretectal nucleus. LT=lateral terminal nucleus of the accessory optic tract. NB=nucleus of the brachium of the inferior colliculus. RN=red nucleus. SPF=subparafascicular nucleus.

**Extended Data Fig. 8:**
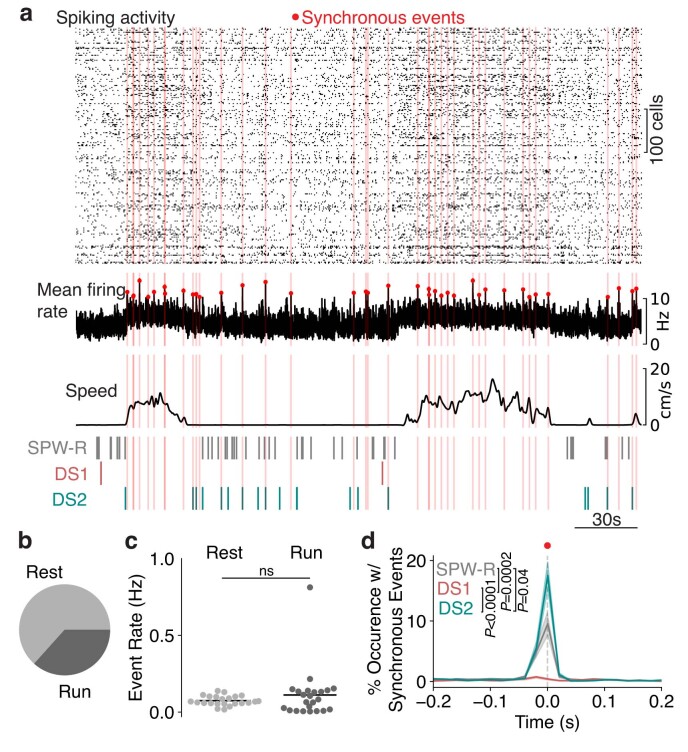
DS2 is most coincident with periods of high brain-wide firing. **a**, Firing rate data averaged across all cells from all brain areas was used to identify peaks (synchronous events) in activity exceeding the 99.9^th^ percentile (red lines and dots), plotted alongside speed and the occurrence of hippocampal LFP events. **b**, Pie chart shows average percent of brain-wide synchronous events (red dots in **a**) with greater (run) or less (rest) than 1 cm/s. **c**, Rate of occurrence of brain-wide synchronous events during rest and run. Paired t-test (two-sided), t_22_ = 0.84, *P* = 0.54. **d**, Average (±sem) rate of occurrence of hippocampal LFP events time-locked to brain-wide synchronous events (red dot at time 0, same as red lines and dots in **a**). Repeated measures ANOVA, F_2,44_ = 26.83, *P* < 0.0001. Two-sided Tukey post-test. Data are from the Allen Brain Institute^31^ and Steinmetz datasets^32^, n = 23 sessions from 20 mice.

As previously reported, average firing rates during SPW-Rs were indeed elevated in hippocampal regions and their direct downstream output targets, such as retrosplenial cortex and lateral septum^22,23^. Most of the recorded brain regions demonstrated higher average firing rates and more recruited units during DS2 than SPW-R, which was virtually ubiquitous throughout the cortex, thalamus and midbrain (Fig. 2a–c,e and Extended Data Fig. 7). Similarly, periods of high brain-wide firing activity were most coincident with DS2 (Extended Data Fig. 8). Firing rates during the less frequent DS1 were much lower than DS2 (Fig. 2a) and rarely occurred with high brain-wide firing (Extended Data Fig. 8), emphasizing the importance of segregating DSs by type. Across many brain areas, units with high firing rates during DS2 were often distinct from those with high firing rates during SPW-R, highlighting a prominent orthogonal relationship in neural activity (Extended Data Fig. 7).

**Extended Data Fig. 9:**
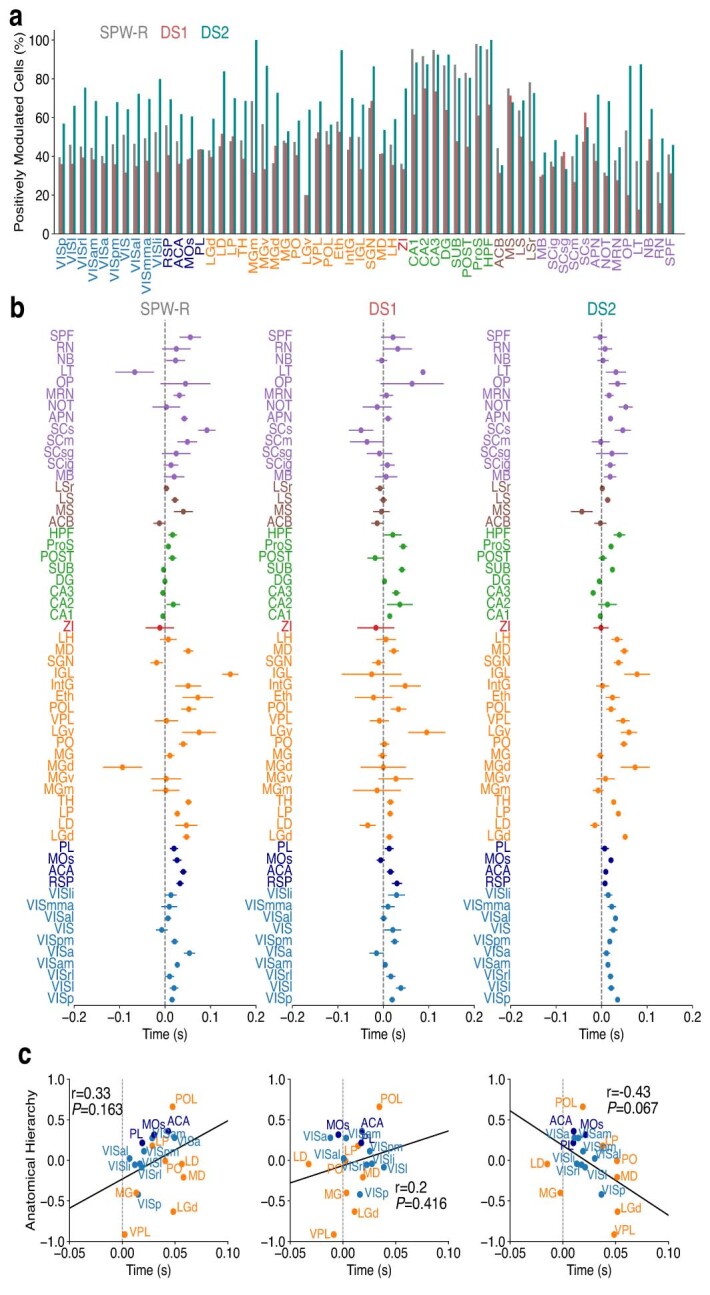
Timing of peak firing changes for positively modulated units. **a**, Percent of cells significantly positively modulated by SPW-R, DS1, or DS2 per brain region (see Methods for shuffling procedures). **b**, Timing of peak firing rate change, relative to event peak, for positively modulated units from **a** (n varies by brain area, see Fig. 2a for total unit numbers per region). Data are mean ± SEM. **c**, Relationship between anatomical hierarchy and the average timing of peak firing rate changes from **b** for each event type. Two-sided Pearson correlation coefficient (r) and *P* value are displayed. Data are from the Allen Brain Institute^31^ and Steinmetz datasets^32^.

Firing rate changes during DS2 were positively corelated to an anatomical hierarchy score (Fig. 2d), which was derived from a detailed anatomical tracing study that sorted corticothalamic areas from low (for example, primary visual) to high (for example, association cortex) on the basis of connectivity patterns^24^. The timing of firing rate peaks across this hierarchy also suggested a possible opposing pattern of activity propagation for SPW-Rs (low to high) versus DS2 (high to low) (Extended Data Fig. 9). To further assess brain-wide coordination, we derived the mutual information between activity changes in pairs of brain regions. The results are shown as a connected graph, showing dense connections and the highest percentage of significantly coupled brain regions for DS2 (Fig. 2f–h). Thus, DS2 and SPW-Rs are associated with fundamentally different brain dynamics, which raises the question of whether these events serve different functions.

## Encoding of current location during DS2

**Fig. 3:**
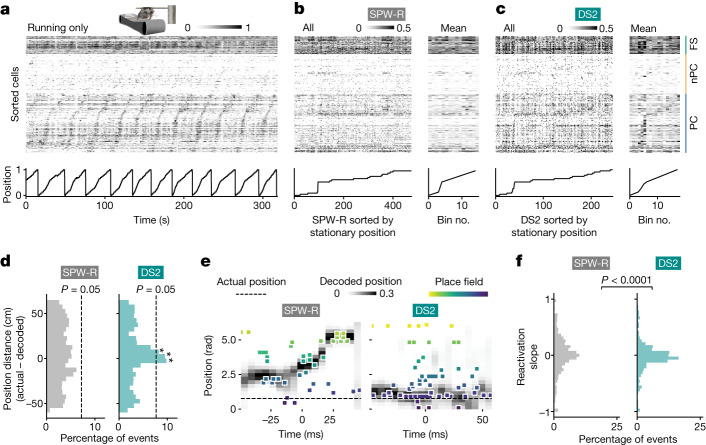
Reactivation of place cells encoding current position during DS2. **a**–**c**, Normalized position along a cued treadmill (bottom panels) and firing rates of place cells (PC, *n* = 117), non-place cells (nPC, *n* = 78) and fast-spiking cells (FS, *n* = 35) in CA1 during locomotion (**a**) and for non-locomotor periods during SPW-Rs (**b**), and DS2 from an example session (**c**). **b**,**c**, The left shows firing rates during individual events, sorted by position where the event occurred along the *x* axis. The right shows averaged firing rates collapsed across spatial bins (note that diagonal tiling of place cell activity matches actual position for DS2, but not SPW-R). **d**, Difference in actual versus decoded (using neural activity from in **b** and **c** as inputs) position for SPW-R and DS2. Decoded positions were circularly shifted randomly 1,000 times for statistical analysis (Methods). *P* = 0.05 cutoff is represented by a vertical line. *P < 0.05, *n* = 7 mice. **e**, Example reactivation sequences during SPW-R (reactivation slope is 0.51) and DS2 (reactivation slope −0.02) showing CA1 place cell firing (squares are individual spikes coloured and sorted along the *y* axis by place field position) and decoded position probability (black intensity) relative to actual position (dotted line). **f**, Slope of a linear fit for decoded reactivation trajectories. Absolute slope values were compared with a Mann–Whitney *U*-test (two-sided). *U* = 162,805, *P* < 0.0001. *n* = 7 mice. Data were collected from the authors’ laboratory.

**Extended Data Fig. 10:**
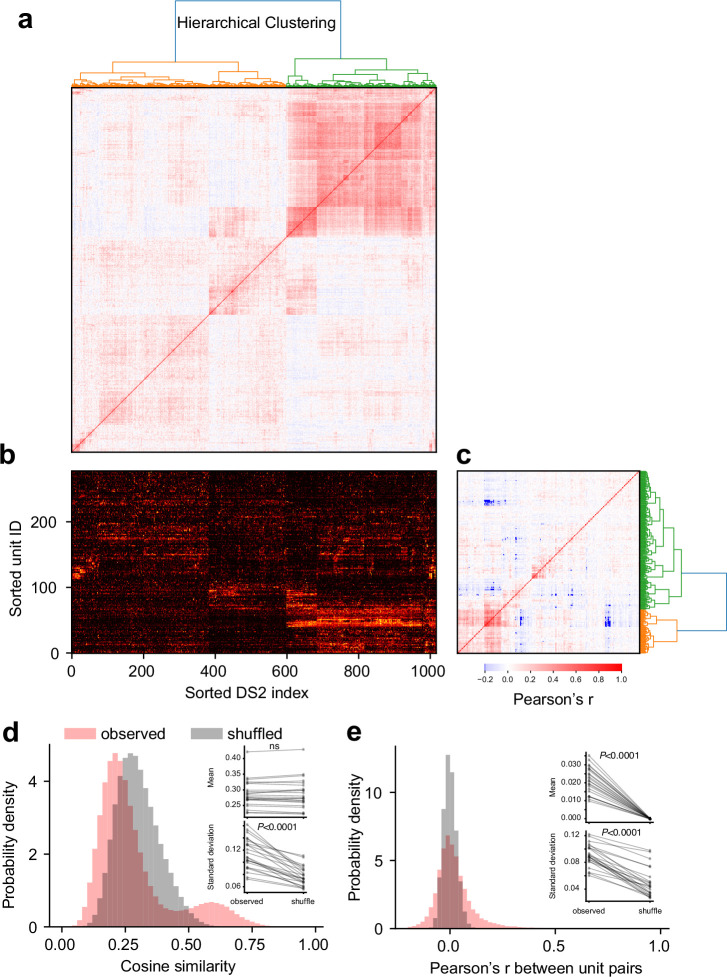
Ensemble activity pattern during DS2. **a**, Correlation matrix using two-sided Pearson’s r for all pairs of population vectors during DS2 from a representative recording session. DS2 indices were sorted using agglomerative hierarchical clustering, shown on the top. Two clusters were identified with a Euclidean distance metric and the Ward variance minimization linkage method. **b**, Normalized brain-wide ensemble activity during DS2 from the same representative recording session. Single units and DS2 events were sorted by agglomerative hierarchical clustering. **c**, Same as in **a**, but for all unit pairs combination. **d**, Similarity of population vectors between individual DS2 events quantified by cosine similarity. Distributions of cosine similarity values from the same representative recording session were shown for the observed and shuffled data in red and gray, respectively. DS2 indices for each unit were shuffled to obtain null distribution. Inset: Mean and standard deviation of cosine similarity from all recording sessions (n = 23, paired two-sided t-test for mean: t_22_ = 0.28, *P* = 0.78; for standard deviation: t_22_ = 9.29). **e**, Same as in **d**, but for Pearson’s r between unit pairs (n = 23, paired two-sided t-test for mean: t_22_ = 14.18; for standard deviation: t_22_ = 12.89). Data are from Allen Brain Institute^31^ and the Steinmetz dataset^32^.

**Extended Data Fig. 11:**
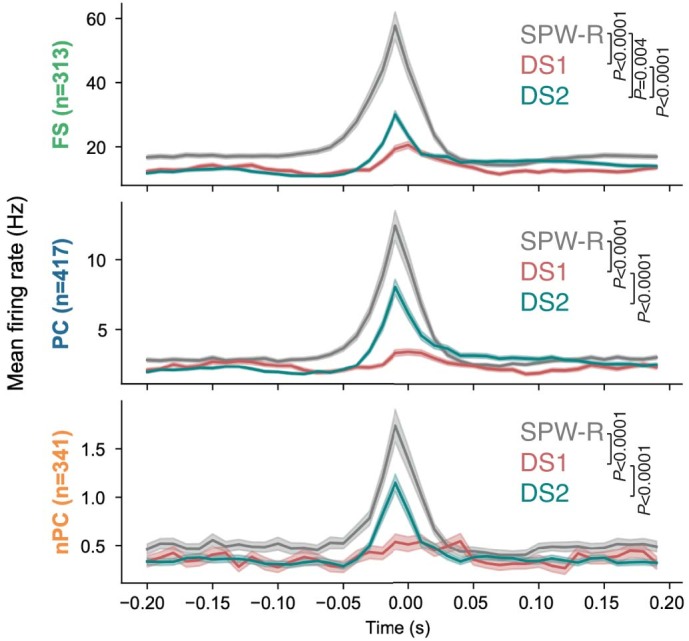
Mean CA1 firing rate during SPW-R, DS1, and DS2. Average (±sem) firing rate surrounding hippocampal LFP events. Firing rates within 40msec of each event and session mean were compared by repeated measures one-way ANOVA was performed for each cell type. F_3,936_ = 57.8, *P* < 0.0001 for FS, F_3,1020_ = 38.6, *P* < 0.0001 for nPC, F_3,1248_ = 49.3, *P* < 0.0001 for PC. Two-sided post-hoc pairwise comparisons with Bonferroni correction. Data were collected in the authors’ lab.

**Extended Data Fig. 12:**
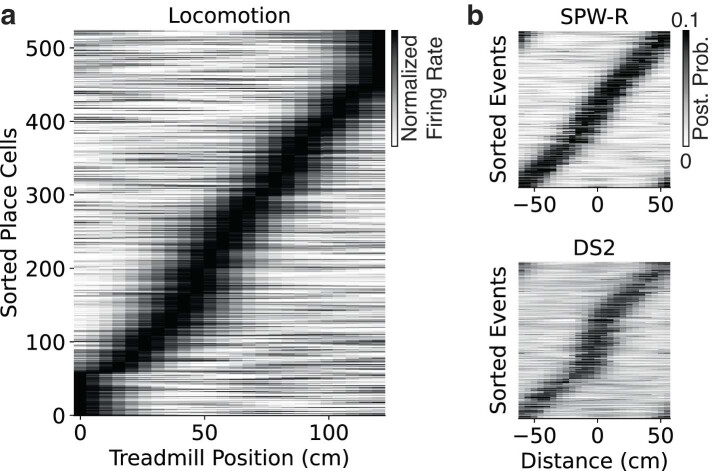
Place cell tuning during locomotion, SPW-R, and DS2. **a**, Normalized firing rates of place cells are sorted by their preferred firing fields (n = 7 mice), showing a homogeneous distribution of place fields tiling the 120-cm track. **b**, Posterior probability of decoded position (black intensity) with respect to the mouse’s current physical location (distance=0 cm) for all SPW-R and DS2 from all mice. Note the overrepresentation of DS2 events with a higher probability of the decoded position aligning to a distance of 0 cm from the mouse’s current location.

**Extended Data Fig. 13:**
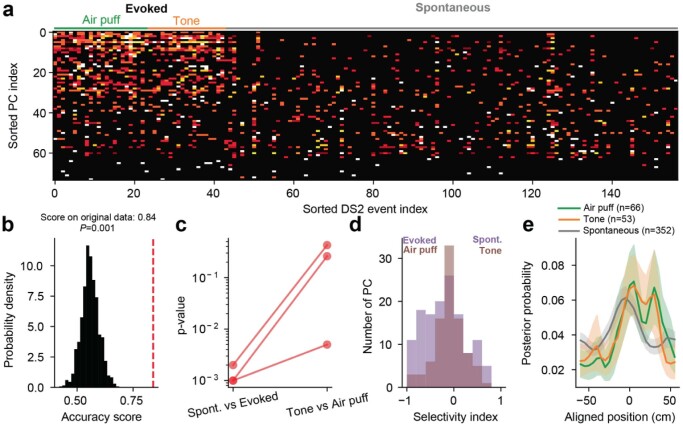
Current position coding is retained for evoked DS2. **a**, Example recording session showing PC activity during various categories of DS2. PCs are sorted by firing preference of evoked DS2 and DS2 events are sorted by their category. Hotter color denotes higher firing rate. **b**, Decoding of evoked or spontaneous DS2 using the PC activity in (**a**). A perceptron was trained to do the classification. The category of DS2 was shuffled 1000 times to generate the null distribution of accuracy scores, shown in black bars. A red vertical dashed line marks the accuracy score on original data (two-sided permutation test). **c**, *P*-values of decoding DS2 category from all mice were obtained using two-sided permutation test. Each dot corresponds to one mouse and the same mouse is connected by a line (n = 3 mice). **d**, Distribution of PC firing preference for different categories of DS2. Selectivity index is defined by the difference of PC mean firing rates during each category of DS2 divided by their sum. Note more PC were recruited to evoked DS2 while most PC had no firing preference for the type of sensory stimuli. **e**, Bayesian decoding of animals’ position from ensemble PC activity during various categories of DS2. Decoded position is aligned to animals’ location. Data are mean (solid colored lines) with the shaded area representing 95 percent bootstrapped confidence interval. Repeated measures ANOVA, F_23,10764_ = 18.2, *P* < 0.0001 main effect of position. Data were collected in the authors’ lab.

It has previously been observed that ensembles of neurons in the dentate gyrus are repeatedly and synchronously active during immobility^25^, which we also observed specifically during DS2 (Extended Data Fig. 10), but the information contained within these ensemble activations is unknown. Place cells fire at unique locations (place fields) in an environment, which are thought to collectively yield a cognitive map for spatial navigation^26^ and gives us the opportunity to determine whether the distinct brain and behavioural state accompanying DS2 supports a different spatial coding scheme from SPW-Rs. During SPW-Rs, place cells with fields distant from the mouse’s actual location are reactivated to represent past or future trajectories (‘replay’)^4–11^. How does the neural representation of place return to the current location and ground the animal to the real-world between replay events? Given the connection of DS2 to brief arousal events, we proposed that hippocampal population activity during DS2 would reflect this re-engagement with current location. To this end, we recorded the spiking activity of large populations of place cells in CA1 and compared spatial coding during the firing rate elevations that accompany DS2 and SPW-R (Extended Data Fig. 11). Indeed, place cells recorded during running were also active during DS2 while mice were immobile at those place cells’ preferred locations, which was not apparent for SPW-Rs (Fig. 3a–c and Extended Data Fig. 12). We quantified this phenomenon by plotting a histogram of distances between actual position in physical space versus the position decoded from neural activity by a Bayesian classifier. Decoded DS2 events reflecting the mouse’s actual position were over-represented in our dataset, reflecting alignment of the neural population activity to the mouse’s real-world location (Fig. 3d). Furthermore, current location encoding was also conserved for evoked DS2, whereas the identity of the sensory stimulus (tone versus puff) was not reliably decoded (Extended Data Fig. 13). Compared to SPW-R, DS2 population activity were more likely to have little or no net change in decoded position over time (Fig. 3e,f). Thus, unlike the offline replay trajectories that include locations distant from the animal’s actual location during SPW-Rs, DS2 is associated with a stationary representation of the mouse’s actual location.

## DS2 supports associative memory

**Fig. 4:**
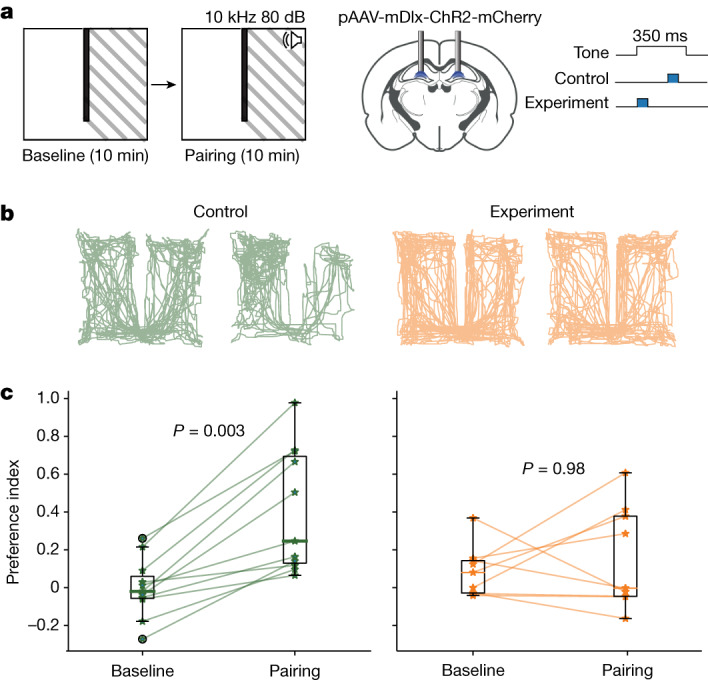
DS2 supports associative memory acquisition. **a**, Schematic of the experimental design, showing a place preference task on the left and optogenetic intervention on the right. **b**, Example trajectories of mice during the two phases of the task. **c**, Place preference during the two phases of the task. Each line denotes one mouse (*n* = 11 control mice, *n* = 9 experiment mice). The box ranges from the first quartile to the third quartile of the data, with a coloured line at the median. The whiskers extend from the box to the farthest data point lying within 1.5 times the interquartile range from the box. Flier points are those past the end of the whiskers. Note by the time the light was delivered in the control group, DS2s were already triggered by the tone. Repeated measures two-way ANOVA, *F*_1,18_ = 8.27, *P* = 0.01, followed by pairwise two-sided multiple comparisons with Bonferroni correction. Data were collected from the authors’ laboratory.

**Extended Data Fig. 14:**
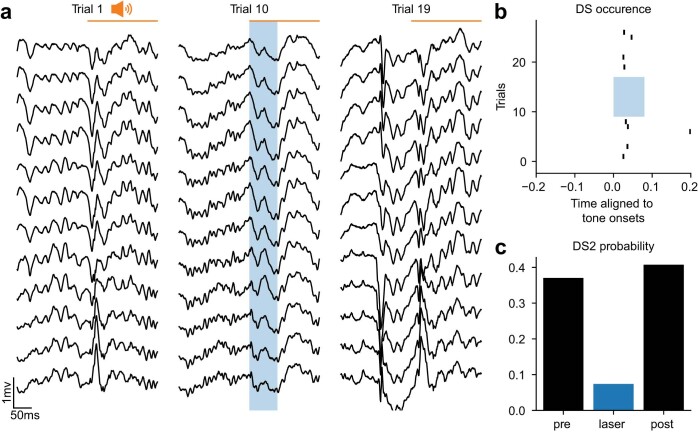
Evoked DS2s silenced by optogenetic activation of DG interneurons. **a**, Raw LFP traces, ordered by recording depth, from representative trials during tone presentation before, during, and after laser stimulation in DG. **b**, A raster plot of detected DS2 aligned to tone onsets from a representative recording session. Blue shaded region indicates laser timing. **c**, Probability of detecting DS2 immediately after tones (0 to 80 ms after tone onsets) before, during, and after laser stimulation in DG (n = 3 mice). Data were collected in the authors’ lab.

**Extended Data Fig. 15:**
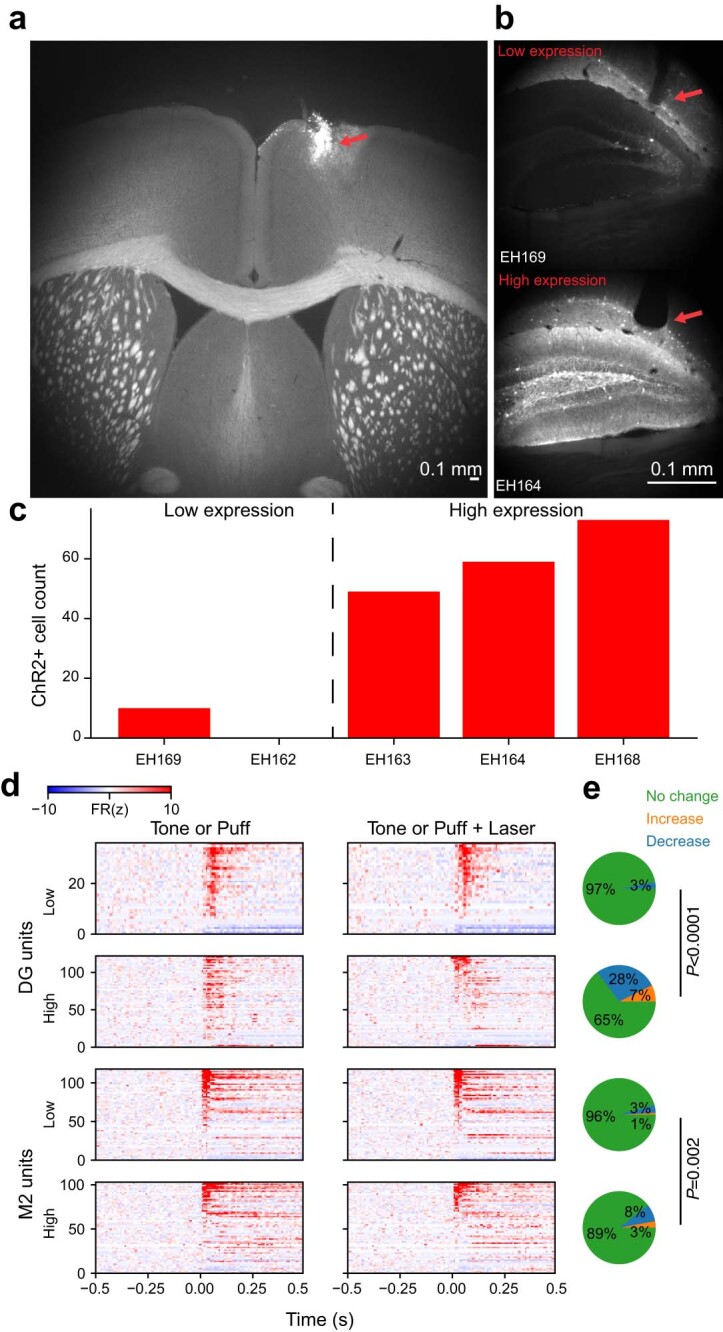
Single unit response to silencing evoked DS2. **a**, A coronal brain slice showing probe locations in secondary motor regions (M2). A red arrow points to the probe track revealed by DiI on the tips of probes. **b**, Channelrhodopsin (ChR2) expression in the DG inhibitory neurons. Upper and lower panels show examples of low and high ChR2 expression, respectively. Red arrows point to locations of optic fiber tips. **c**, Number of ChR2-positive cells in the DG for each mouse. Cells were counted from two consecutive 60-μm brain slices with the deepest optic fiber track. Mice with cell count lower than 20 were considered low ChR2 expression. **d**, Normalized firing rate of single units in DG and M2 aligned to sensory stimulus onsets. Units were separated into low and high ChR2 expression. Right and left panels show unit response to sensory stimulus with and without laser stimulation. **e**, Proportion of single units that significantly change their firing rates with laser stimulation. Trial indices were shuffled 1000 times to generate null distribution of normalized firing rate for each unit. Mean normalized firing rates were taken from 0 to 0.08 s after the onset of sensory stimuli. Units with mean normalized firing rate greater or smaller than 99th or 1st percentile of the null distribution (Monte Carlo’s p value less than 0.01) during the trials with laser were considered significantly increased or decreased by laser stimulation, respectively. Proportions of each type of units (i.e., no change, increase, and decrease) were significantly different between virus expression levels for each brain region (two-sided Chi-square test for DG: X2 (2, n = 122) = inf, for M2: X2 (2, n = 103) = 12.8). Data were collected in the authors’ lab.

Because the elevated neural activity during DS2 contains spatial information, we asked whether this packaging of information serves a potential memory function. We leveraged the fact that DS2 is reliably evoked by a tone within a narrow time window (Fig. 1i–k), enabling temporally precise optogenetic interventions to silence DS2. We used a conditioned place aversion task in which a loud tone is paired with one side of a two-chamber environment (Fig. 4a). In the experimental group, we optogenetically activated dentate GABAergic neurons bilaterally using pAAV-mDlx-ChR2-mCherry within 80 ms of tone onset to silence neural activity during DS2, but did not intervene for the remaining 270 ms of the 350 ms tone presentation. As a control, we inhibited neural activity for the same duration during tone presentation, but outside the time window when DS2 occurs, to leave tone-evoked DS2 intact. We confirmed that this optogenetic approach potently inhibits DS2 (Extended Data Fig. 14) and local dentate unit spiking, and even suppresses spiking activity in cortex (Extended Data Fig. 15). Control mice reliably avoided the side paired with an aversive tone, whereas mice that received precisely timed optogenetic intervention to silence DS2 did not discriminate between the two chambers (Fig. 4b,c). Thus, neural activity during the brief time window when DS2 occurs supports the acquisition of an associative memory.

## Discussion

Here we show that DS2 is an online, synchronous population event accompanied by widespread increases in neural activity and brief arousal transitions. On the basis of the stationary activation of place cells with fields close to the mouse’s current location, we propose that DS2 may serve as a mechanism to regularly ground the hippocampal representation of position in an environment during immobility. Rapidly switching between current (DS2) and remote (SPW-R) locations would enable cognitive flexibility that varies with sudden changes in the animal’s internal state or changes in the environment (for example, a startling noise). Synchronous neural activity during DS2 may provide opportunity windows for synaptic plasticity, consistent with our findings linking DS2 to associative memory formation.

At the microcircuit level, distinct brain states are shaped by the non-uniform recruitment of local inhibitory cells, which are key for directing information flow^27^. As arousal-activated AACs heterogeneously innervate principal cells^19,20^ and are highly active during DS2 but mostly silent during SPW-Rs, this GABAergic cell (and probably others, such as TORO cells) may be important in regulating the distinct ensemble activity between DS2 and SPW-Rs. At a network level, DS2 is thought to be primarily triggered by the medial entorhinal cortex, which contains neurons that encode self-referenced movement variables, locations and environmental borders^28–30^. This self-referenced spatial input may indeed be key for recruiting spatially tuned hippocampal cells corresponding to an animal’s current position during DS2. Both tones and air puffs reliably evoked DS2 and promoted current position encoding, but the identity of the stimulus (tone versus puff) could not be reliably decoded. This may not be surprising considering that primary sensory areas are among the least active brain areas during DS2. Moreover, the short latency between DS2 and stimulus onset points towards faster mechanisms for DS2 generation that probably depend on arousal circuitry. This is in line with the observation that DS2 also occurs during spontaneous changes in arousal, independent of changes in the environment.

Together, these data shed new light on a poorly understood brain and behavioural state, opening the door for a more complete understanding of how neural activity is organized to support memory and cognitive functions. By supporting a more behaviourally engaged brain state that organizes immediately relevant spatial information, DS2 provides a complementary function to the offline nature of SPW-Rs and extends the range of computational possibilities during immobility.

## Methods

### Mice

For datasets generated in this manuscript, all procedures performed were approved by the Administrative Panel on Laboratory Animal Care at Stanford University. Mice were group housed with littermates and kept on a 12/12 h light/dark cycle and at a temperature of 20–25 °C and 30–70% humidity. Experiments were performed during the light phase on male and female mice of 3–8 months of age. C57BL/6J mice were bred in house from mice obtained from Jax (strain no. 00664). Unc5b-2A-Cre^ERT2^ mice were generously provided by J. Huang at Duke University^20^.

### Publicly available Neuropixel recordings

Data from the Allen Brain Institute^31^ can be accessed using the Allen SDK (https://allensdk.readthedocs.io/en/latest/). Data from the Functional Connectivity dataset were used, which included long segments without visual stimuli (wild-type mice only: ten males aged 108–142 days, three females aged 126 to 134 days). Sessions without pupil data were excluded. Data from the Steinmetz study^32^ were obtained from https://figshare.com/articles/dataset/Dataset_from_Steinmetz_et_al_2019/9598406 and included ten sessions from seven mice (five females, aged 12 to 20 weeks, genotypes included tetO-G6s × CaMK-tTA, Vglut1-IRES2-Cre-D, RCL-GCaMP6f × VGlut-Cre, Snap25-GCaMP6s; two males, aged 26 to 30 weeks, genotypes included C57BL/6J and RCL-GCaMP6f × VGlut-Cre). Only sessions with electrodes intersecting the CA1 and dentate gyrus were included in the analysis. For 22 out of 23 recording sessions, the same electrode used for identifying DSs was used for ripple detection to avoid location-specific biases.

### DS and SWR detection

For DS detection, the dentate gyrus was identified by first using the provided anatomical locations of each channel, which are registered to the Allen Mouse Brain Common Coordinate Framework (https://atlas.brain-map.org/)^33^ and easily visualized with the QuickNII tool (https://github.com/HumanBrainProject/QuickNII)^34^. The QuickNII tool was used to generate images of electrode locations on coronal slices. Full probe tracks were visualized with the Brainrender graphical user interface (https://github.com/brainglobe/brainrender)^35^. The channel containing maximal positive spikes during immobility was identified visually and used for identifying spikes. Voltage signals from this channel were bandpass filtered (5–100 Hz) and peaks exceeding 4.5 standard deviations of this filtered signal were identified (4 s.d. was used for Moniz_2017-05-15). The timepoint at the maximum of each peak was considered the DS time and the filtered trace was used for amplitude and half-width quantification. To separate putative spikes into distinct types, we performed standard current source density (CSD) analysis (https://github.com/espenhgn/iCSD)^36^ on channels from the deepest dentate channel to the hippocampal fissure (channel with maximal theta amplitude), followed by principal components analysis on the normalized CSD profile, and then used DBSCAN to cluster the first two principal components into event types. Two clusters representing DS1 and DS2 reliably emerged with this approach. On average, 1.3 ± 0.3% detected spikes were outside these two clusters and were not investigated. Both principal components analysis and DBSCAN analysis were done using the Scikit-learn Python package (https://scikit-learn.org/stable/).

For SWR detection, we performed detection as done previously^21^ and in agreement with consensus in the field^37^. Briefly, voltage signals from the CA1 pyramidal cell layer, determined from ripple and theta power by depth plots and visual inspection, were bandpass filtered (120–180 Hz) and the envelope was derived from the Hilbert transformation. Peaks in the envelope reaching five standard deviations and exceeding three standard deviations for at least 25 ms were included and the maximum positive value of the filtered trace is taken as the ripple time.

### Neuropixel recording analysis

Timestamps for SPW-Rs, DS1 and DS2 were used to collect spiking activity of neurons from 200 ms before and after the event. The minority of events that include multiple event types within 200 ms were excluded from this analysis. Pupil diameter and locomotor speed aligned to each event type were only taken from the Allen Institute data, which permitted forwards and backwards locomotion (note the task-specific perpendicular orientation of the running wheel in the Steinmetz data) and avoided pupil-related changes due to screen illumination differences across the recording. Pupil and speed data in Fig. 1e,f are normalized to the maximum and minimum values observed across the entire session. Event rates and percentage coincidence were reported from Allen Institute data, but similar results were obtained from the Steinmetz data. For each unit, firing rates within an event were obtained by binning at 10 ms intervals over the 400 ms peri-event time, centred on DS peak or ripple envelope peak. Firing rates within an event were then standardized (*z* score) to each unit’s standard deviation and mean firing rate across the entire session, which was obtained from 100 ms bins and smoothed with a Gaussian filter (sigma value of five) as performed previously^38^. Peak firing rate changes were taken as the maximal *z* score from this peri-event firing rate. TORO cells were identified as previously described^21^. To determine the percentage of cells significantly modulated by each event type, peri-event spikes from each cell were randomly shuffled 100 times per event to generate a null distribution. Significant positive modulation was reported if the maximal firing rate bin exceeded the 99.5th percentile of the shuffled distribution. Cells exceeding this cutoff were then used to determine the relative timing of peak firing, averaged within a given brain area.

To identify time-points with high brain-wide firing rates, we binned all spiking activity, averaged across all cells, into 10 ms bins. We then collected timestamps for any peak in this averaged spiking activity that exceeded 99.9th percentile and plotted the event rates during immobility (less than 1 cm s^−1^) or wheel movement (greater than 1 cm s^−1^). We then examined the event-triggered average rate of occurrence for SPW-R, DS2 and DS1 aligned to these periods of high brain-wide firing and performed summary statistics on LFP events (that is, SPW-R, DS2 and DS1) falling within 60 ms of an identified high brain-wide firing event. TORO cells were identified as previously described.

To examine the occurrence of SPW-R, DS2 and DS1 aligned to auditory and visual stimulation, we used the Steinmetz dataset, which replayed task related auditory noises, including tone cues, white noise and clicking of the valve during reward delivery, as well as visual stimuli during a non-task period later in the session (that is, repeatedly played individually without the full behavioural paradigm and without reward to serve as a control).

To determine the percentage of each LFP event with significant increases in gaze movement, we used the Allen Institute data that reliably tracked changes in pupil position (that is, speed). For each event, we derived the change in pupil speed by subtracting the average pupil speed during and after (20 ms before to 85 ms after) from the pupil speed before the event (290 to 85 ms before). For facial movements, we performed the same analyses, but on Steinmetz data (note that there were no facial videos from Allen Institute) and used time windows of 40 to 115 ms after versus 140 to 40 ms before. Each event was randomly shuffled by ±2 s to derive a null distribution. Randomly shuffled events were pooled and the 97.5th percentile (two-sided, *P* < 0.05) was set as the cutoff for a significant increase in pupil or facial movements.

*t*-distributed stochastic neighbour embedding (*t*-SNE)^39^ was also used to visualize high-dimensional variability in spiking patterns across the brain in two-dimensional space, as it relates to facial or pupil movements. Peri-event firing rates from all cells across all events were reduced to an average firing rate (that is, averaged across the 400 ms peri-event time), normalized and embedded in *t*-SNE space (Perplexity 20). A change in pupil or facial movements were obtained for each event and normalized to visualize how motor movements couple to event types along the two *t*-SNE dimensions.

### Mutual information

All data from the Steinmetz and Allen Brain Institute recordings were pooled for this analysis. Mutual information was computed using Python code adapted from the Neuroscience Information Theory Toolbox (https://github.com/nmtimme/Neuroscience-Information-Theory-Toolbox, ref. ^40^). Briefly, spiking data from a ±200 ms time window around SPW-R or DS peaks were sorted into 5 ms bins and discretized into states using four uniform count bins for each brain region. The mutual information, defined by

$$\sum _{x\in {\bf{X}},\,y\in {\bf{Y}}}P(x,y){\log }_{2}\left(\frac{P(x,y)}{P(x)P(\,y)}\right),$$

where *x* and *y* represent spiking activity in different brain regions, *P*(*x*,*y*) is the joint distribution, and *P*(*x*) and *P*(*y*) are the marginal distributions, was then computed for each time bin. Baseline mutual information values, defined as the mean mutual information values from −200 to −100 ms relative to the event peaks, were subtracted to obtain Δmutual information for each brain region pair. Maximum Δmutual information values were used to construct the graphs using the NetworkX Python package (https://networkx.org). To determine the significance of mutual information estimation, event trials were shuffled 5,000 times to obtain a null distribution and a Monte Carlo *P* value less than 0.01 was considered significant. Only brain region pairs with at least five sessions were included.

### Two-photon imaging of AACs

Surgical procedures, two-photon imaging and analysis of AAC calcium were performed as described previously^20^. Briefly, adeno-associated viral (AAV) particles for the calcium indicators GCaMP6f (AAVDJ-CAMKII-GCaMP6f; UNC, deposited by K. Deisseroth)^41^ and jRGECO1a (AAV1-FLEX-jRGECO1a)^42^ were injected into three Unc5b-2A-Cre^ERT2^ mice (two females, one male) and surgically implanted with a 3 mm imaging cannula. pAAV.Syn.Flex.NES-jRGECO1a.WPRE.SV40 was a gift from D. Kim and the GENIE Project (Addgene viral prep no. 100853-AAV1; RRID:Addgene_100853). To induce Cre-dependent expression, mice were anaesthetized with isoflurane and injected intraperitoneally with tamoxifen (100 mg kg^−1^, Sigma) dissolved in corn oil (Sigma) at 20 mg ml^−1^ on days 2, 5 and 8 after virus injection. Following recovery, a 16-channel silicon probe (A1x16-3mm-50-177-H16_21mm, NeuroNexus) was inserted into the contralateral hemisphere under electrophysiological guidance to identify SPW-Rs and DSs during subsequent recordings. SPW-R and DS detection were performed as above (‘Neuropixel recording analysis’ section) and from voltage signals collected using the same recording system as discussed below (‘Place cell electrophysiological recordings from head-fixed mice’ section). Imaging data were collected at 15.49 frames per second on a two-photon microscope (Neurolabware) equipped with a ×16 objective (0.8 numerical aperture (NA), Nikon WI) using 1,000 nm excitation from a tuneable Ti:Sa laser (MaiTai, Spectra Physics) whereas locomotor movements were tracked with a rotary encoder equipped to a treadmill and facial movements were captured from a video camera (Mako, Allied Vision) and quantified by deriving the frame by frame motion energy map on a cropped region containing the face, as previously described^20^. Data were synchronized to the electrophysiological recording (https://open-ephys.org) to align AAC calcium changes (jRGECO1a) and facial movements to DSs and SPW-Rs, or sensory stimulation events. In separate sessions, auditory tones (80 dB, 10 kHz, 0.35 s) and air puffs (five PSI, 0.1 s, delivered 5 cm from the face, 45° left of the nose), ten trials of each, were delivered in an alternating manner with a random interval between 15 and 45 s. Hardware delays were measured for each stimulus and used to accurately measure evoked DS latency. Motion correction was performed and regions of interest were detected using SIMA^43^. Unc5b-AAC regions of interest were segmented using the STICA method in SIMA^43^, and cells that were not detected by this step were manually added by drawing a region around the soma. Other neurons in the pyramidal layer (putative pyramidal cells) were segmented using the PlaneCA1PC method of SIMA. Δ*F*/*F* traces were computed using a third-order polynomial fit as the time-dependent baseline. Calcium changes (Δ*F*/*F*) for sensory stimulation or electrophysiological events were reported as mean *z*-scores across events and/or trials and cells, using the preceding 1 s of data for standardization.

### Place cell electrophysiological recordings from head-fixed mice

Seven mice (five females, two males) on a C57BL/6J background were implanted with metal head bars on their skulls using Super Glue and dental cement. Two coordinates were marked with black ink on the skull for craniotomies later (−2 mm anteroposterior and ±2 mm mediolateral relative to bregma). After 1 week of recovery, mice were head restrained on a linear treadmill for at least three 10 min daily sessions until they were comfortable enough to initiate movement spontaneously on the track. On the day before the acute recording experiment, craniotomies were performed near the marked coordinates. Cranial windows were then sealed with silicone (Kwik-Cast, WPI) whenever mice were not in a recording experiment. On the day of acute recording experiment, silicone was removed from awake head-restrained mice and the exposed cranial windows were filled with saline. Micromanipulators (MPC-200, Sutter Instruments) were used to slowly lower silicon probes with 32 (A1x32-6mm-50-177, NeuroNexus) or 128 channels (128J, UCLA probe^44^ into the right dentate gyrus and left CA1, respectively. The recording sites were confirmed by the presence of DS and SPW-R. Raw signals were amplified and digitized at 30 kHz by Intan headstages connected to an open ephys acquisition system (https://open-ephys.org). Spikes from single units were sorted using Kilosort (https://github.com/MouseLand/Kilosort), followed by manual curation in Phy2 (https://github.com/cortex-lab/phy). Treadmill movement was determined by a quadrature rotary encoder on one of the axles of the two wheels supporting a fabric belt. Each lap was detected when a neodymium magnet on a fabric belt passed through a hall sensor (KY-003, MXRS) fixed on a treadmill. Rotations and magnetic signals were recorded by analogue to digital converters and synchronized with electrophysiological recordings. In three mice, random air puff or tone presentations, as in the ‘Two-photon imaging of AACs’ section, were delivered at 10 s intervals.

### Bilateral SPW-R and DS recordings

Surgical procedures, as in the ‘Place cell electrophysiological recordings from head-fixed mice’ section, were performed on five mice (three females, two males). Two linear probes (A1x32-6mm-50-177, NeuroNexus) were slowly inserted into each hippocampus until DSs were visually identified. Then 30 min recordings were obtained from mice as they behaved on a floating Styrofoam ball. Bilaterally occurring SPW-Rs and DSs were considered synchronous if they occurred within 100 ms. The reference probe was the probe with fewer SPW-Rs or DSs. A subsequent 10 min recording was obtained during random air puff or tone presentation, as in the ‘Two-photon imaging of AACs’ section, at 10 s intervals. Movement of the Styrofoam ball as previously described^38^ was used to identify periods of locomotion and immobility (0.5 cm s^−1^ cutoff) by taking the average ball speed 1 s before tone and/or puff onset.

### Bayesian decoding of position

A Bayesian decoding algorithm was implemented to translate ensemble spiking activity near SPW-R and DS2 into angular positions on the treadmill^45^. Position information on the 120-cm-long treadmill was discretized into 5 cm bins. The probability of a mouse being at location *x*, given the number of spikes from each unit recorded in a 20 ms time window, was estimated by Bayes’ rule: *P*(*x*|*n*) = (*P*(*n*|*x*) × *P*(*x*))/(*P*(*n*)), where *P*(*n*|*x*) was approximated from the position tuning of each place unit under the assumption that the number of spikes from each unit followed a Poisson distribution and the position tuning of individual units was statistically independent. Previous knowledge of position, *P*(*x*), was set to 1 to prevent decoding bias to any position on the treadmill. The normalizing constant, *P*(*n*), was set to ensure the sum of posterior probability, or *P*(*x*|*n*), equal to 1. The position bin with the highest probability was taken as the decoded position. A decoding error was the distance between decoded and actual positions. To determine whether any decoding error bin was over-represented, decoded positions were circularly shifted with random values drawn from −60 to 60 for 1,000 times. From each shuffling iteration, the maximum proportion of decoding error distribution was taken to determine global significance level. Any decoding error bins with proportion above the 97.5th percentile of the shuffled maximum proportions (7.7 and 8.4% for SPW-R and DS2, respectively) were considered over-represented. To detect candidate replay sequence events during SPW-R and DS, smoothed multi-unit activity (MUA) in successive 1 ms time window had to exceed mean MUA. MUA was smoothed by convolving the raw spike count with a 21 ms Hanning window. To remove smoothing artefact, event boundary was adjusted inwards so that at least one spike was included in the first and last time bins. Candidate events with duration less than 50 ms or more than 2 s were excluded from further analysis. Next, ensemble activity within each candidate event was decoded using the Bayesian method mentioned above in a sliding 20 ms time window that shifted 5 ms at each step. Slopes of decoded trajectories were estimated by performing a circular-linear regression on the posterior probability distribution^46^. Slopes with goodness-of-fit (*R*^2^) values of less than 0.3 were excluded. Bayesian decoding analyses were performed using custom codes written in Python.

### Decoding of stimulus identity for evoked DS2

A perceptron was trained to classify DS2 events (evoked versus spontaneous or tone versus air puff) on the basis of firing rate vectors of place cells using Scikit-learn Python package (https://scikit-learn.org/stable/index.html). Stratified tenfold cross validation (‘StratifiedKFold’ function) was performed on the dataset. To evaluate the significance of an accuracy score for the original data, a permutation test (‘permutation_test_score’ function) shuffling the identity of sensory stimuli for 1,000 times was used to generate a null distribution of accuracy scores. An empirical *P* value was then calculated as the percentage of permutations for which the score obtained was greater that the score obtained using the original data.

### Similarity analysis of brain-wide ensembles during DS2

Population activity vectors were defined by the number of action potentials from each unit within a 200 ms time window centred at the peak of each DS2 event and normalized by the maximum number of action potentials within each unit. Units with mean firing rate above 5 Hz or without any action potentials during DS2 were excluded from this analysis. Pearson’s correlation coefficients and cosine similarity were then computed for all pairs of normalized population vectors during DS2. To arrange units and DS2 events on the basis of similarity of their activity pattern, agglomerative hierarchical clustering algorithm with a Euclidean distance metric and the Ward variance minimization linkage method was implemented using Scipy Python package (https://scipy.org/). Statistical significances of Pearson’s correlation coefficients and cosine similarity between pairs of population vectors were assessed by shuffling DS2 indices within each unit.

### Real-time place preference task

Twenty C57BL6/J mice (nine females, 11 males) with ages ranging from 3 to 4 months old were used at the time of behavioural testing. To broadly silence network activity, we targeted an excitatory opsin to GABAergic cells. To this end, mice first received stereotactic injection of 400 nl of pAAV-mDlx-ChR2-mCherry bilaterally (−2.3 mm anteroposterior, ±1.5 mm mediolateral, −2 mm dorsoventral relative to bregma), followed by implantation of 200-μm-core-diameter optic fibres (−2.3 mm anteroposterior, ±1.5 mm mediolateral, −1.6 mm dorsoventral relative to bregma). The implanted mice were given at least 2 weeks for recovery and virus expression. To acclimate mice to movement with overhanging fibres before behaviour testing, they were trained to freely explore a clear acrylic behaviour box (32 × 22.5 × 22.5 cm) with optic fibres attached for at least three 20 min daily sessions. On the day of behaviour testing, mice were placed in a white opaque acrylic behaviour box (31 × 24.5 × 22 cm) with a black opaque barrier (21.5 × 19 × 3 cm) partially separating the box into two compartments. To make the two compartments visually distinctive, one compartment’s side wall was decorated with vertical stripes using black tape and the other compartment’s side wall was left blank. To establish a baseline preference for each compartment, mice were free to explore both compartments without any experimental intervention for the first 10 min. During the subsequent 10 min, a loud tone (80 dB, 10 kHz, 350 ms) was played from a niobium speaker (Power Acoustik) placed in the behaviour box every 10 s whenever mice entered the stimulation compartment. A blue laser (5 mW, 80 ms, 430–490 nm) was delivered following the onsets of tones with time delays of 0 and 250 ms for mice in the experimental and control groups, respectively. The head position of mice was tracked in real-time using an overhead web camera (Logitech, 30 frames per s) running custom scripts on Bonsai (https://bonsai-rx.org/) with a pretrained model from DeepLabCut (https://deeplabcut.github.io/DeepLabCut/README.html). Once the mouse’s head entered the stimulation compartment, the custom Bonsai program delivered transistor–transistor logic (TTL) signals to an Arduino microcontroller (https://www.arduino.cc/) to trigger tones and the laser. In separate experiments, three more mice underwent procedures similar to Bilateral SPW-R and DS recordings, but an optrode (A1x32-Poly3-10mm-50-177 with 105-μm-core-diameter optical fibre from Neuronexus) was lowered into the hippocampus instead of a linear probe. Light was delivered as above to determine whether evoked DS2 could be silenced.

### Electrophysiological recordings from head-fixed mice during silencing of evoked DS2

Surgical procedures described in the ‘Real-time place preference task’ section were performed on five female mice, except only the left hemisphere received optic fibre implant targeting the dentate gyrus. Furthermore, dentate gyrus (−2.3 mm anteroposterior, +1.5 mm mediolateral relative to bregma) and M2 (+0.5 to +1.5 mm anteroposterior, +0.7 mm mediolateral relative to bregma) coordinates were marked for subsequent acute recordings. A four-shanks NeuroNexus silicon probe with a 200 μm core and 0.5 NA optic fibre attached (A4x32-Poly2-5mm-20s-150-160-OAC128) was inserted into dentate gyrus or M2 in separated recording sessions when the mice were head restrained on a cued linear treadmill. A loud tone (80 dB, 10 kHz, 0.35 s) or air puff (five PSI, 0.5 s) directed to each mouse’s body was presented with or without blue laser (5 mW, 80 ms, 430–490 nm) delivered to both hemispheres at 0.1 Hz. Spike sorting was performed as described in the ‘Place cell electrophysiological recordings from head-fixed mice’ section. Probe locations were verified post hoc by Dil staining on 60 μm coronal brain sections.

### Statistics

All statistical tests were performed using Python and can be found in the figure legends, unless otherwise stated. For analysis of variance (ANOVA), post hoc *P* values were adjusted for multiple comparisons.

### Reporting summary

Further information on research design is available in the Nature Portfolio Reporting Summary linked to this article.

## Online content

Any methods, additional references, Nature Portfolio reporting summaries, source data, extended data, supplementary information, acknowledgements, peer review information; details of author contributions and competing interests; and statements of data and code availability are available at 10.1038/s41586-024-07192-8.

## Supplementary information

Reporting Summary

## Data Availability

Neuropixel data are publicly available from the Allen Institute (https://allensdk.readthedocs.io/en/latest/)^31^ and Nicholas Steinmetz (https://figshare.com/articles/dataset/Dataset_from_Steinmetz_et_al_2019/9598406)^32^. Other data from this study are available from the corresponding author on reasonable request.

## Code availability

The code used in this study is indicated in the Methods.
