# Supplementary material for: Neural and behavioural state switching during hippocampal dentate spikes
Source: Nature. 2024 Mar 13;628(8008):590–5. doi: 10.1038/s41586-024-07192-8 (PMC11023929; doi:10.1038/s41586-024-07192-8)
Supplement: Supplementary file 1 — Reporting Summary [file 41586_2024_7192_MOESM1_ESM.pdf]

nature portfolio

Corresponding author(s): Jordan Farrell

Last updated by author(s): Dec 5, 2023

Reporting Summary

Nature Portfolio wishes to improve the reproducibility of the work that we publish. This form provides structure for consistency and transparency in reporting. For further information on Nature Portfolio policies, see our Editorial Policies and the Editorial Policy Checklist.

Statistics

For all statistical analyses, confirm that the following items are present in the figure legend, table legend, main text, or Methods section.

n/a	Confirmed	
☐	☒	The exact sample size (*n*) for each experimental group/condition, given as a discrete number and unit of measurement
☐	☒	A statement on whether measurements were taken from distinct samples or whether the same sample was measured repeatedly
☐	☒	The statistical test(s) used AND whether they are one- or two-sided<br>*Only common tests should be described solely by name; describe more complex techniques in the Methods section.*
☐	☒	A description of all covariates tested
☐	☒	A description of any assumptions or corrections, such as tests of normality and adjustment for multiple comparisons
☐	☒	A full description of the statistical parameters including central tendency (e.g. means) or other basic estimates (e.g. regression coefficient) AND variation (e.g. standard deviation) or associated estimates of uncertainty (e.g. confidence intervals)
☐	☒	For null hypothesis testing, the test statistic (e.g. *F*, *t*, *r*) with confidence intervals, effect sizes, degrees of freedom and *P* value noted<br>*Give P values as exact values whenever suitable.*
☐	☒	For Bayesian analysis, information on the choice of priors and Markov chain Monte Carlo settings
☒	☐	For hierarchical and complex designs, identification of the appropriate level for tests and full reporting of outcomes
☒	☐	Estimates of effect sizes (e.g. Cohen's *d*, Pearson's *r*), indicating how they were calculated

Our web collection on statistics for biologists contains articles on many of the points above.

Software and code

Policy information about availability of computer code

Data collection	The open ephys GUI (https://openephys.org) and Bonsai v2.8 (https://bonsai-rx.org/) were used to collect electrophysiological and behavioral data, respectively.
Data analysis	Code from the following openly available tools were used for data analysis: DeepLabCut (https://deeplabcut.github.io/DeepLabCut/README.html), Scipy (https://scipy.org/), Scikitlearn (https://scikit-learn.org/stable/index.html), Phy2 (https://github.com/cortex-lab/phy), Kilosort (https://github.com/MouseLand/Kilosort), NetworkX python package (https://networkx.org), Neuroscience Information Theory Toolbox (https://github.com/nmtimme/Neuroscience-Information-Theory-Toolbox), current source density (CSD) analysis (https://github.com/espenhgn/iCSD), Brainrender GUI (https://github.com/brainglobe/brainrender), QuickNII tool (https://github.com/HumanBrainProject/QuickNII), Allen Mouse Brain Common Coordinate Framework (https://atlas.brain-map.org/).

For manuscripts utilizing custom algorithms or software that are central to the research but not yet described in published literature, software must be made available to editors and reviewers. We strongly encourage code deposition in a community repository (e.g. GitHub). See the Nature Portfolio guidelines for submitting code & software for further information.

Data

Policy information about availability of data

All manuscripts must include a data availability statement. This statement should provide the following information, where applicable:

- Accession codes, unique identifiers, or web links for publicly available datasets
- A description of any restrictions on data availability
- For clinical datasets or third party data, please ensure that the statement adheres to our policy

Neuropixel data are publicly available from the Allen Institute (https://allensdk.readthedocs.io/en/latest/) 22 and Nicholas Steinmetz (https://figshare.com/articles/dataset/Dataset_from_Steinmetz_et_al_2019/9598406) 26. Other data from this study are available from the corresponding author on reasonable request.

Research involving human participants, their data, or biological material

Policy information about studies with human participants or human data. See also policy information about sex, gender (identity/presentation), and sexual orientation and race, ethnicity and racism.

Reporting on sex and gender	N/A
Reporting on race, ethnicity, or other socially relevant groupings	N/A
Population characteristics	N/A
Recruitment	N/A
Ethics oversight	N/A

Note that full information on the approval of the study protocol must also be provided in the manuscript.

Field-specific reporting

Please select the one below that is the best fit for your research. If you are not sure, read the appropriate sections before making your selection.

☒ Life sciences ☐ Behavioural & social sciences ☐ Ecological, evolutionary & environmental sciences

For a reference copy of the document with all sections, see nature.com/documents/nr-reporting-summary-flat.pdf

Life sciences study design

All studies must disclose on these points even when the disclosure is negative.

Sample size	Sample size was informed by the authors' prior experience performing similar electrophysiological, behavioral, and 2-photon imaging experiments (see references 20, 21, and 38).
Data exclusions	No data was excluded from the manuscript.
Replication	Increased CA1 firing during DS2 was verified in all three datasets (Authors', Steinmetz, and Allen Institute). Other experiments were performed once.
Randomization	Experimental and control mice were pulled randomly from cages of littermates and assigned after surgery, ensuring that housing conditions and surgical interventions were balanced across groups.
Blinding	Blinding was not necessary for experiments involving the analysis of neural activity and behavioral correlates of hippocampal LFP patterns. For Figure 4, mouse behavioral analysis was automated to prevent bias.

Reporting for specific materials, systems and methods

We require information from authors about some types of materials, experimental systems and methods used in many studies. Here, indicate whether each material, system or method listed is relevant to your study. If you are not sure if a list item applies to your research, read the appropriate section before selecting a response.

Materials & experimental systems

n/a	Involved in the study
☒	☐ Antibodies
☒	☐ Eukaryotic cell lines
☒	☐ Palaeontology and archaeology
☐	☒ Animals and other organisms
☒	☐ Clinical data
☒	☐ Dual use research of concern
☒	☐ Plants

Methods

n/a	Involved in the study
☒	☐ ChIP-seq
☒	☐ Flow cytometry
☒	☐ MRI-based neuroimaging

Animals and other research organisms

Policy information about studies involving animals; ARRIVE guidelines recommended for reporting animal research, and Sex and Gender in Research

Laboratory animals	Mice were group housed with littermates and kept on a 12:12 light:dark cycle and at a temperature of 20-25C and 30-70% humidity. Experiments were performed during the light phase on male and female mice of 3-8 months of age. C57BL/6J mice were bred in house from mice obtained from Jax (strain #00664). Unc5b-2A-CreERT2 mice were generously provided by Josh Huang at Duke University
Wild animals	No wild animals were used in this study.
Reporting on sex	Data were collected from both sexes. Group sizes were not sufficiently large enough to assess sex differences.
Field-collected samples	No field collected samples were used in the study.
Ethics oversight	For datasets generated by the authors, all procedures were approved by the Administrative Panel on Laboratory Animal Care at Stanford University.

Note that full information on the approval of the study protocol must also be provided in the manuscript.

Plants

Seed stocks	N/A
Novel plant genotypes	N/A
Authentication	N/A
